# On the Use of the Electro-Magnet in Removing Particles of Iron from the Eye

**Published:** 1883-03

**Authors:** Lucien Howe


### On the Use of the Electro-Magnet in Removing Particles of Iron from the Eye.

By Lucien Howe, M. D.

It was one of the dreams of older ophthalmic surgeons that fragments of iron or steel, when firmly imbedded in the cornea or in the sclerotic, might be removed by means of a magnet. This appears, at first glance, so plausible, that by many it is supposed to be an easy matter, and it is not unusual for a laborer who has been thus injured to enter a physician's office expecting magic relief by some such means. Unfortunately the fragments are so small as to be attracted only slightly, even by a strong magnet; and, moreover, they are frequently imbedded so firmly in the cornea or other portion as to require considerable force to dislodge them. Until recently, therefore, the method was regarded as practically useless in every instance.

During the last five or six years, however, a number of cases have been reported, in which the magnet served a good purpose in the removal of such foreign bodies, when lodged in the vitreous humor. Easily movable as they were in this gelatinous substance, they could be directly drawn to a certain spot on the sclerotic, and then removed through an opening there made, although usually with some escape of the vitreous. If it were desirable to cite such examples, I might add to the list by detailing an operation of this kind, in which the magnet proved almost invaluable.

But my object at present is to give the outlines of two cases, one of which shows that the magnet is useful in dislodging such particles from the cornea; the other suggests an improvement in the ordinary methods of removing them from within the globe.

The first, was that of a laborer, D. K., who, when striking with a hammer on a flinty rock, suddenly felt something enter his left eye. A physician who was consulted, failed to notice any special cause for the pain of which the man complained, and, considering the injury to be a slight abrasion, dismissed him with a prescription for an astringent lotion. Some slight injection, lachrymation and pain persisted, and on the 17th of December, 1882, thirty-eight days after the accident, the patient applied to me.

I found, at that time, in addition to the symptoms mentioned, a small dark speck on the outer part of the cornea opposite the edge of the pupil. Oblique illumination revealed this to be the end of a fragment of iron which subsequent measurements showed to be about one and one-half millimetre long and one-half millimetre broad. It had evidently struck the cornea with the point foremost, and passing inward, about one-half remained in that structure, while the other half projected into the anterior chamber. The point of entrance was partly closed, thus causing comparatively little pain or irritation.

All efforts to remove the particle in the ordinary manner, by means of a needle, were painful and fruitless. On disturbing the end which was imbedded in the cornea, the other end could be seen to move in the aqueous humor. To persist in such attempts would be to run the risk of dislodging the piece and pushing it into the globe against the lens, producing cataract, or on to the iris, giving rise to severe inflammation. It would have been also dangerous to attempt to pass a knife in behind it, and push it outwards. To remove it with forceps of any kind was impossible.

There seemed to be only one solution of the difficulty, only one method which gave any hope of immediate relief. It was to draw out the fragment by means of a magnet. Fortunately this means of assistance was at hand, in the form of the electro-magnet, described by Dr. Conrad Frolich in the *Klinische Monatsblätter für Augenheilkunde* for 1881, and manufactured by Hess, of Berlin. The eye had become so sensitive, from efforts at removal with a needle, and photophobia was so great, that it was deemed worth while to administer an anæsthetic. Having done this, the point of entry in the cornea was first enlarged, and then the pole of the electro-magnet placed over the fragment. Instantaneously it was dislodged, a spurt of aqueous following its exit, as it came out attached to the point of the instrument.

This case seems worthy of mentioning,

1st. Because of the peculiar position of the fragment and the consequent difficulties in its removal.

2d. Because the electro-magnet was useful in really *extracting* a particle of iron from the cornea—an application of that instrument which has seldom or never before been made.

The second case was that of a machinist, F. D., from Dunkirk, N. Y., who, while at work on the driving-wheel of an engine, was struck by a particle of iron on the left eye. He came under my observation on the day following the accident, December 13, 1881. At that time he suffered no pain or other inconvenience, but there was slight ciliary injection, a small wound in the upper part of the cornea could be detected, and immediately beneath this a glistening fragment, which measured, subsequently, two millimetres by three-fourths of a millimetre. This had not penetrated the lens, but simply rested with the sharp point near the edge of the iris, as was shown by the ready dilatation of the pupil when atropia was used. The question as to how it should be removed was not an easy one to answer. Any ordinary instrument, when intruded into the anterior chamber, would be apt to tip the fragment over upon the lens, thus wounding the capsule and producing cataract; or, there was danger of a similar effect by attempting an iridectomy in the usual manner. In other words, it appeared eminently advisable, whenever the anterior chamber was punctured, that the fragment should be drawn out at once through the opening—that it should not be pressed between the posterior surface of the cornea and the anterior surface of the lens, or, at least, that the iris be kept interposed between the sharp edges of the iron and the lens, in order to protect the latter.

With a view to accomplishing this, the handle was cut off oí an ordinary iridectomy knife, and the blade screwed firmly into the helix of the electro-magnet, already referred to. The patient having been etherized by his family physician, Dr. J. M. McWharf, an opening was made at the edge of the cornea, in the usual manner for performing an iridectomy. As soon as the magnetized knife approached the fragment, the latter bent over and attached itself to the point of the instrument. But unfortunately for the perfection of the operation, when the knife was withdrawn through the narrow opening the fragment of iron was scraped off, as it were, and still remained in the interior of the the globe, partly hidden in the folds of the iris. Every attempt to extract the fragment alone was unsuccessful, and it was therefore necessary to draw out a large part of the iris surrounding it, and cut it off.

The wound healed readily, and the only disadvantageous effect remaining was the irregular form of the pupil, the inevitable result of the iridectomy.

The case presents three points oí interest:

1st. The position of the fragment was unusual, such particles being arrested much more frequently by some resisting tunic of the eye than by the soft tissue of the iris.

2d. The use of a magnetized knife in this locality has not before been recommended, at least to my knowledge, and the good purpose which it served, up to a certain stage, shows that the suggestion is worthy of some little consideration.

3d. The fact that the particle was wiped off from the knife as the latter was being withdrawn through the edges of the wound shows the necessity of modifying this part of all similar operations, no matter in what part of the eye the foreign body is located. In other words, it is desirable that at the moment of exit the wound be made wider to facilitate the withdrawal of the instrument with the attached fragment, as could be accomplished, for example, by dilating the wound at that instant with a pair of fine forceps.

The result obtained in this second case might be considered satisfactory, and the method of operating at least safer than that formerly employed. But reflection upon the details of the procedure shows that greater improvement might have been made. To point out these, for the benefit of other operators, seems of more practical use than simply to relate the history of a fortunate case.

